# Degenerative Pathways of Lumbar Motion Segments - A Comparison in Two Samples of Patients with Persistent Low Back Pain

**DOI:** 10.1371/journal.pone.0146998

**Published:** 2016-01-25

**Authors:** Rikke K. Jensen, Per Kjaer, Tue S. Jensen, Hanne Albert, Peter Kent

**Affiliations:** 1 Research Department, Spine Centre of Southern Denmark, Hospital Lillebaelt, Institute of Regional Health Research, University of Southern Denmark, Middelfart, Denmark; 2 Department of Sports Science and Clinical Biomechanics, University of Southern Denmark, Odense, Denmark; 3 Department of Orthopaedics, Bartholomew's Hospital, London, England; Brown University, UNITED STATES

## Abstract

**Background:**

Magnetic resonance imaging (MRI) is used to identify spinal pathoanatomy in people with persistent low back pain. However, the clinical relevance of spinal degenerative MRI findings remains uncertain. Although multiple MRI findings are almost always present at the same time, research into the association with clinical outcomes (such as pain) has predominantly focused on individual MRI findings. This study aimed to: (i) investigate how multiple MRI lumbar spine findings cluster together within two different samples of patients with low back pain, (ii) classify these clusters into hypothetical pathways of degeneration based on scientific knowledge of disco-vertebral degeneration, and (iii) compare these clusters and degenerative pathways between samples.

**Methods:**

We performed a secondary cross-sectional analysis on two dissimilar MRI samples collected in a hospital department: (1) data from the spinal MRI reports of 4,162 low back pain patients and (2) data from an MRI research protocol of 631 low back pain patients. Latent Class Analysis was used in both samples to cluster MRI findings from lumbar motion segments. Using content analysis, each cluster was then categorised into hypothetical pathways of degeneration.

**Results:**

Six clusters of MRI findings were identified in each of the two samples. The content of the clusters in the two samples displayed some differences but had the same overall pattern of MRI findings. Although the hypothetical degenerative pathways identified in the two samples were not identical, the overall pattern of increasing degeneration within the pathways was the same.

**Conclusions:**

It was expected that different clusters could emerge from different samples, however, when organised into hypothetical pathways of degeneration, the overall pattern of increasing degeneration was similar and biologically plausible. This evidence of reproducibility suggests that Latent Class Analysis may provide a new approach to investigating the relationship between MRI findings and clinically important characteristics such as pain and activity limitation.

## Background

Magnetic resonance imaging (MRI) is often used clinically to identify serious spinal pathoanatomy in people with persistent low back pain (LBP). However, little is known about the clinical relevance of patterns of other spinal MRI pathological or degenerative findings in clinical populations[1] and such findings have displayed a mostly weak and inconsistent association with the presence of pain in the general population[2].

In a 2011 review, Endean et al. [2] investigated the association between LBP and various MRI findings. The authors concluded that although LBP was only weakly associated with the individual MRI findings they investigated, it was possible that combinations of MRI findings might be more strongly associated. Cheung and colleagues (2009)[3] also investigated this, reporting a positive correlation between the sum of degenerative disc MRI findings and LBP. Similarly, a recent study by Kovac et al.[4] reported that a statistically significant positive association between LBP and severe disc degeneration disappeared when adjusting for other MRI findings, such as Modic changes (vertebral endplate signal changes) and disc protrusion/herniation. These studies support the notion that the co-existence of multiple MRI findings is potentially important when investigating the association between MRI findings and LBP.

Furthermore, there is normally no way of differentiating age-related degeneration (‘biological age’) from trauma-related degeneration on MRI or histology[5], suggesting that the biological process is the same in normal aging but is accelerated in the pathological state. Therefore, the biological age of a vertebral motion segment is not necessarily the same as the actual chronological age of the person.

An additional step in advancing this area of investigation would be to explore how various MRI findings group or cluster together. In a previous study[6], we investigated if there were methods to better model the multivariable relationships between clusters of MRI findings, as these might provide a clearer understanding of how degenerative processes are expressed across all the structures associated with a vertebral segment. We found that Latent Class Analysis could be used to identify clusters of MRI findings in people with LBP and that those clusters could be grouped into biologically plausible hypothetical degenerative pathways that had face validity.

However, the reproducibility of biological pathways identified using this method needed to be tested. If such degenerative pathways are broadly reproducible across MRI samples, they may form a stable platform to investigate the relationship between degenerative pathways and clinically important characteristics such as pain and activity limitation.

Therefore, the aims of this study were to apply this Latent Class Analysis method to two different samples of similar patients with persistent LBP to: (i) determine how multiple MRI findings from lumbar spine motion segments cluster together within each sample, (ii) investigate if these clusters could be classified into hypothetical pathways of degeneration based on the face validity of known histological changes of disco-vertebral degeneration within each sample, and (iii) compare the clusters and pathways across samples.

## Method

### Study sample

This study is a secondary analysis of two different MRI samples (described below) named ‘MRI_1’ and ‘MRI_2’. Both samples were of patients with persistent LBP referred to the same secondary hospital setting (Spine Centre of Southern Denmark), from general practitioners and chiropractors in primary care.

MRI_1: These data were drawn from the total consecutive series of MRI narrative reports generated by the Spine Centre between the years 2000 and 2008. Detailed description of the selection procedure, including the MRI protocol, has been reported elsewhere[7]. In brief, of the 5,919 consecutive MRI narrative reports available, 1,757 (29.7%) were excluded due to: the MRI scan having been performed on the thoracic or cervical spine, it being a repeat MRI scan on the same individual, the scan date not being reported, it lacked identification data or it being a duplicate. Therefore, the final sample size for this sample was 4,162 individual MRI reports (20,810 lumbar motion segments). The mean age of participating patients was 46 years (SD 14, range 12–92) and 51% were female. Information on spinal pathology from the narrative reports, generated by one of two experienced radiologists, was extracted and coded (categorical data) into an electronic coding matrix by three research assistants. The inter-rater reliability of this quantitative coding ranged from substantial to almost perfect agreement (kappa range 0.74–1.00)[8].

MRI_2: These data were from a cohort of patients who had been screened as potential participants in a randomised controlled trial[9], using a protocol that has been previously reported[6,9]. In brief, from June 2006 to June 2008, all patients at the Spine Centre who met the following inclusion criteria were referred for MRI as part of this screening procedure: (a) LBP or leg pain of 3 or more on an 11-point Numerical Rating Scale, (b) duration of current symptoms from 2 to 12 months, and (c) age above 18 years. The MRIs were quantitatively coded using a detailed, standardised research MRI evaluation protocol[10,11] by an experienced musculoskeletal research radiologist who was blinded to any participant information other than name, age and sex. Previous testing of the MRI evaluation protocol by the same radiologist had shown moderate to almost perfect agreement for inter- and intra-observer reliability (kappa range 0.59–1.0) [10–12]. The final sample size for this sample was 631 individual MRI reports (3,155 lumbar motion segments). The mean age of participating patients was 42 years (SD 10.8, range 18–73) and 54% were women.

### MRI variables of interest

To enable comparison in this study between the two samples, only MRI variables common to both samples were selected. The results of clusters derived from 11 dichotomous variables (disc bulge, disc degeneration, disc herniation, facet joint degeneration, High Intensity Zone in the disc (HIZ), Modic changes Type1, Modic changes Type 2, osteophytes, nerve root compromise, spondylolisthesis, spinal stenosis) were compared between samples. Some recoding of variables was initially required to harmonise their content.

MRI_1: The variables in this sample were binary (‘present’ or ‘not present’) and included intervertebral disc bulge, intervertebral disc degeneration, intervertebral disc herniation, facet joint degeneration, high intensity zone (HIZ), Modic changes Type 1 and Modic changes Type 2, osteophytes, nerve root compromise, spondylolisthesis (antero- or retrolisthesis) with or without spondylosis, and spinal stenosis.

MRI_2: This sample had binary as well as categorical variables with more subcategories. To match the structure of MRI_1, the categorical variables were dichotomised into binary variables using the arbitrary, but clinically intuitive, methods described below.

Intervertebral disc degeneration was a dichotomous variable created from two disc-related variables: disc signal intensity and disc height. Disc signal intensity had four categories: 1) hyper-intense; 2) hyper-intense with visible intra-nuclear cleft; 3) intermediate signal intensity; 4) hypo-intense. Disc height also had four categories: 1) disc higher than the disc above; 2) disc as high as the disc above (if normal); 3) disc narrower than the disc above (if normal); 4) endplates almost in contacts. So an intervertebral disc was categorised as being degenerated if its disc signal intensity was Category 3 or 4 and/or its disc height was Category 3 or 4.

Disc herniation had the following categories: 1) no protrusion; 2) focal protrusion; 3) broad-based protrusion; 4) extrusion and 5) sequestration. Disc herniation was dichotomised into no herniation (Category 1) or herniation (Categories 2–5).

Facet joint degeneration was a binary variable (present or not present) regardless of whether or not the finding was present on the left, right or both sides.

Modic changes Type 1 and Type 2 were coded if present at the upper and/or lower endplate in a vertebral motion segment. If both types of Modic changes were present in the same endplate or at the same vertebral level (two endplates), it was coded as a Type 1 change present and a Type 2 change present. Modic changes Type 3 were not coded.

Nerve root compromise had four categories: 1) no contact with nerve root; 2) in contact with nerve root; 3) dislocation of nerve root; 4) compression of nerve root. Nerve root compromise was coded as present, regardless of location, if this variable was Category 2, 3 or 4.

A vertebral segment was categorised as having spondylolisthesis if antero- or retrolisthesis were present with a Meyerding[13] grade of 1 or more.

Spinal stenosis could be central, foraminal or lateral recess. It had three categories: 1) normal; 2) relative or 3) severe. Spinal stenosis was coded as present if any of the variables for the three anatomical areas (central, foraminal or lateral recess) were Category 2 or 3.

### Statistical analyses

As an individual person’s lumbar motion segments can have varying stages of degeneration (segments displaying varying ‘biological age’ despite a person’s ‘chronological age’), the analyses were made at the level of the vertebral segments. Every person in the study contributed with five lumbar vertebral motion segments to the analysis.

A comparison of the prevalence of MRI findings within the individual variables in the two samples was performed using a two-sample test of proportions using STATA 12.0 (StataCorp LP, College Station, Texas, USA).

Clusters of MRI findings were identified in both samples using the multivariable procedure Latent Class Modelling in the statistical program Latent Gold (version 4.5, Statistical Innovations, Belmont MA, USA). The default settings of the software were used for this analysis. In Latent Class Analysis, our identification of the optimal number of clusters used only Schwarz’s Bayesian Information Criterion, which is a method for finding the cluster solution that explains the most variance while requiring the simplest specification of the model.

We used the cluster membership for each vertebral motion segment to determine the proportion of vertebral segments within each cluster that displayed the presence of an MRI finding and the proportion of vertebral levels (L1/2, L2/3, L3/4 L4/5 and L5/S1) within each cluster. These data were calculated and graphed using Excel 2003 (Microsoft Corporation, Redmond, WA, USA).

Using content analysis, each cluster was then categorised by the co-authors into hypothetical pathways of degeneration based on the face validity of known histological changes of disco-vertebral degeneration. These histological changes had previously been identified by an (unpublished) electronic literature search (PubMed and Medline) and review. The pathways were identified independently by the first author and then independently by a group of three co-authors. Consensus was reached by discussion if different pathways were identified. A post-hoc calculation of the mean (SD) chronological age of the motion segments within each cluster was also performed to test if the chronological age of each cluster challenged or supported the concept of ‘biological age’ inherent in the pathways.

### Ethics statement

As this was a secondary analysis of de-identified existing data collected for other research purposes, under Danish law these analyses did not require separate ethics approval. The original studies complied with the Declaration of Helsinki (2008) and were approved by the Institutional Review Board (MRI_1 and MRI_2) and the Ethics Committee for the Region of Southern Denmark (approval # S-VF-20060111) (MRI_2).

## Results

### Individual MRI findings

The prevalence of individual MRI findings on all eleven included variables in the two samples ranged between 1% and 46% (median 5% MRI_1, 12% MRI_2). There were statistically significant differences between the two samples (MRI_1 and MRI_2) in the prevalence of MRI findings in all variables (Table 1). Almost all of the MRI findings, except for spondylolisthesis, were more prevalent in MRI_2 than in MRI_1. Although the absolute difference was often small (median 4%, range 2% to 16%), the relative difference (the highest prevalence in either sample divided by the absolute difference between the samples) was often quite large (median 50%, range 13% to 83%).

**Table 1:** A comparison of the prevalence of individual MRI findings in sample MRI_1 and MRI_2.

Variable	MRI_1	MRI_2	P-value
Disc bulge	24%	39%	<0.00
Disc degeneration	40%	46%	<0.00
Disc herniation	13%	15%	<0.01
Facet joint degeneration	4%	8%	<0.00
High intensity zone	3%	18%	<0.00
Modic changes Type1	5%	9%	<0.00
Modic changes Type2	3%	7%	<0.00
Osteophytes	7%	23%	<0.00
Nerve root compression	4%	12%	<0.00
Spondylolisthesis	3%	1%	<0.00
Spinal stenosis	5%	7%	<0.00

### Clusters of MRI findings

Latent Class Analysis identified six clusters of MRI findings in both samples. Each cluster and its distribution of MRI findings is shown diagrammatically in Figs 1, 2, 3, 4, 5, 6, 7, 8, 9, 10, 11 and 12.

**Fig 1:**
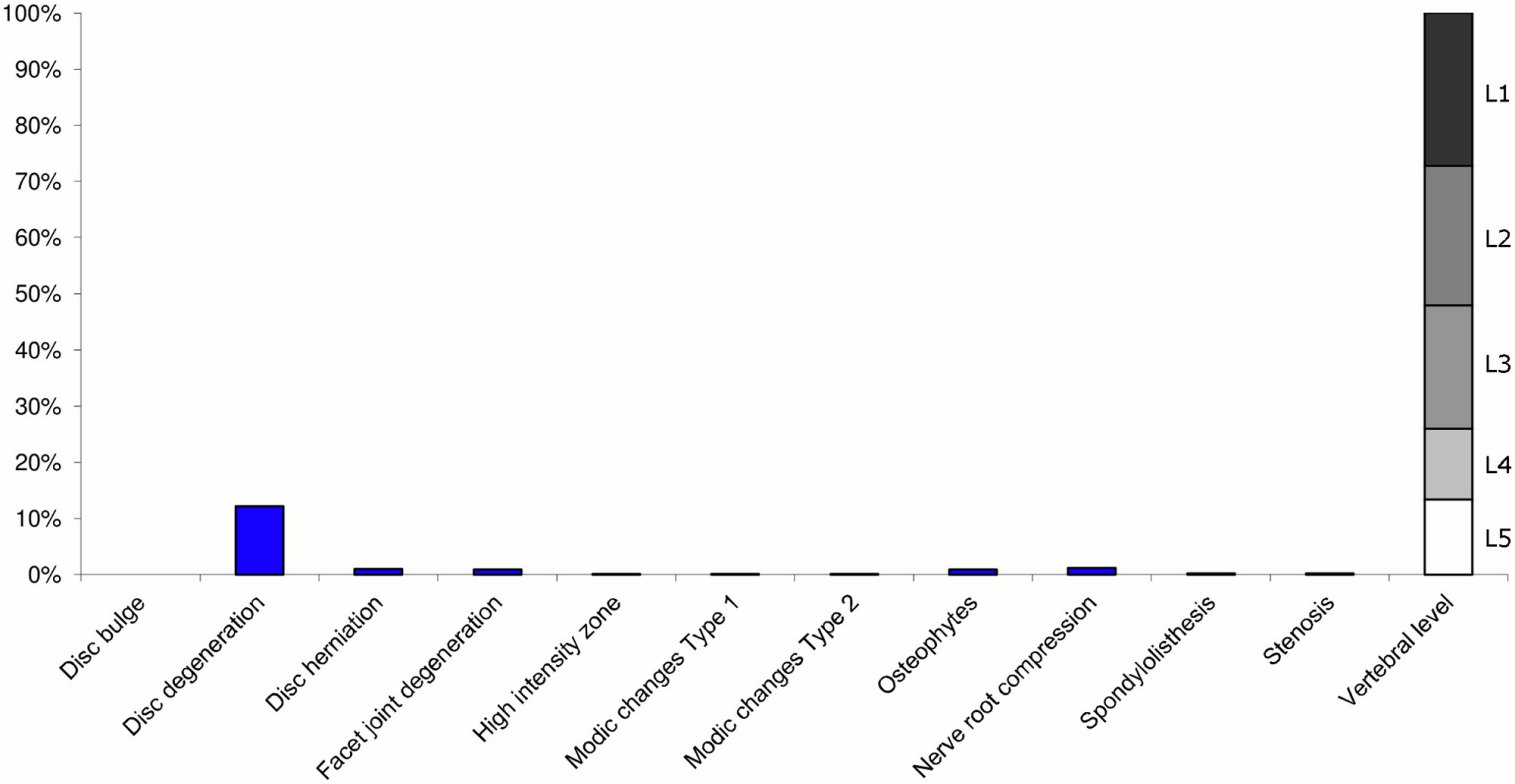
Prevalence of MRI findings within Cluster 1.1 in MRI_1. This cluster contained 65% of the 20,810 vertebral motion segments. (a) The vertical bars on the graph represent the proportion of vertebral motion segments for each of the MRI pathologies. (b) The mean age of the motion segments was 44 years (SD 13). (c) The vertebral level indicator shows the relative proportion of vertebral levels (L1 to L5) within the cluster.

**Fig 2:**
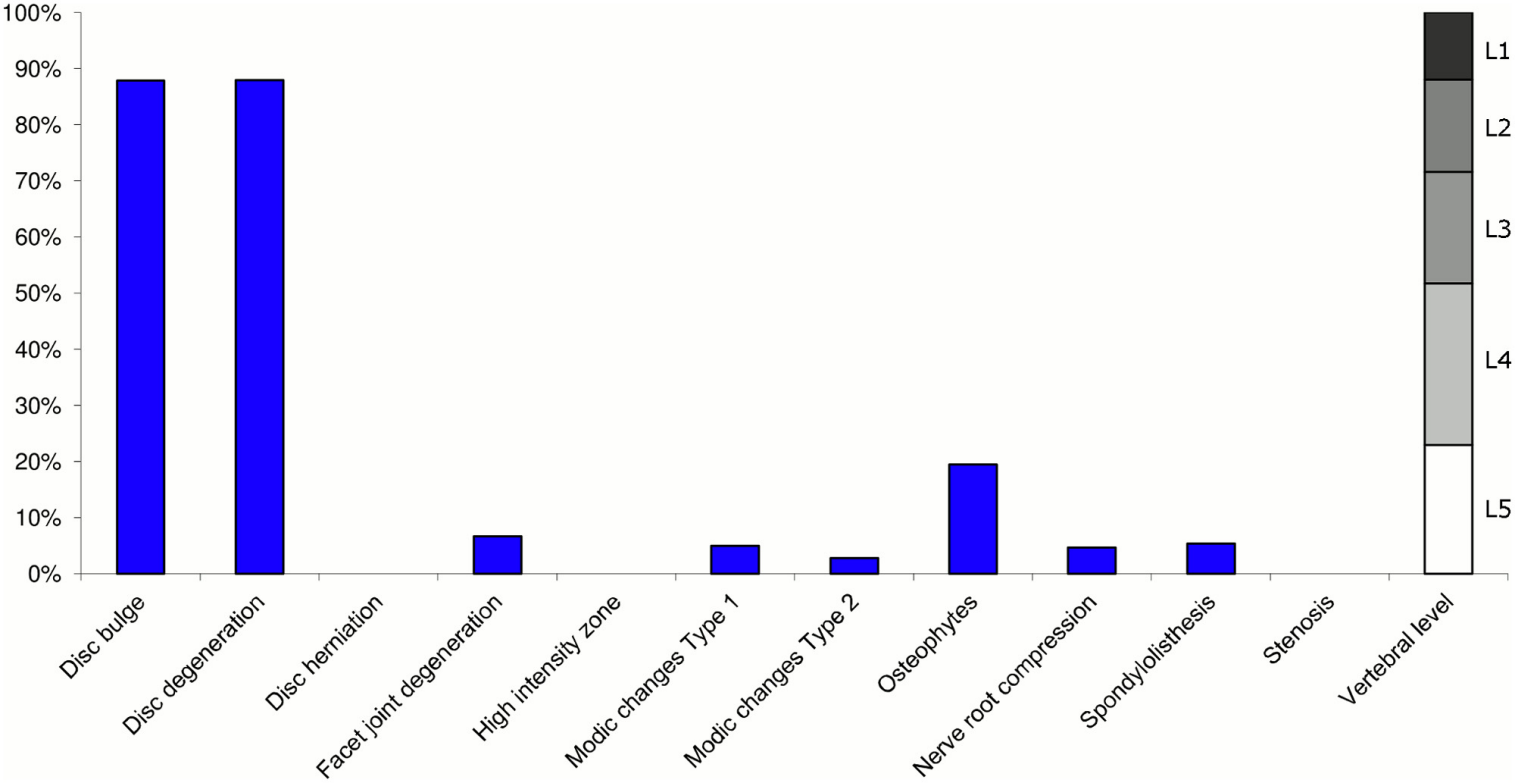
Prevalence of MRI findings within Cluster 1.2 in MRI_1. This cluster contained 16% of the 20,810 vertebral motion segments. (a) The vertical bars on the graph represent the proportion of vertebral motion segments for each of the MRI pathologies. (b) The mean age of the motion segments was 51 years (SD 13). (c) The vertebral level indicator shows the relative proportion of vertebral levels (L1 to L5) within the cluster.

**Fig 3:**
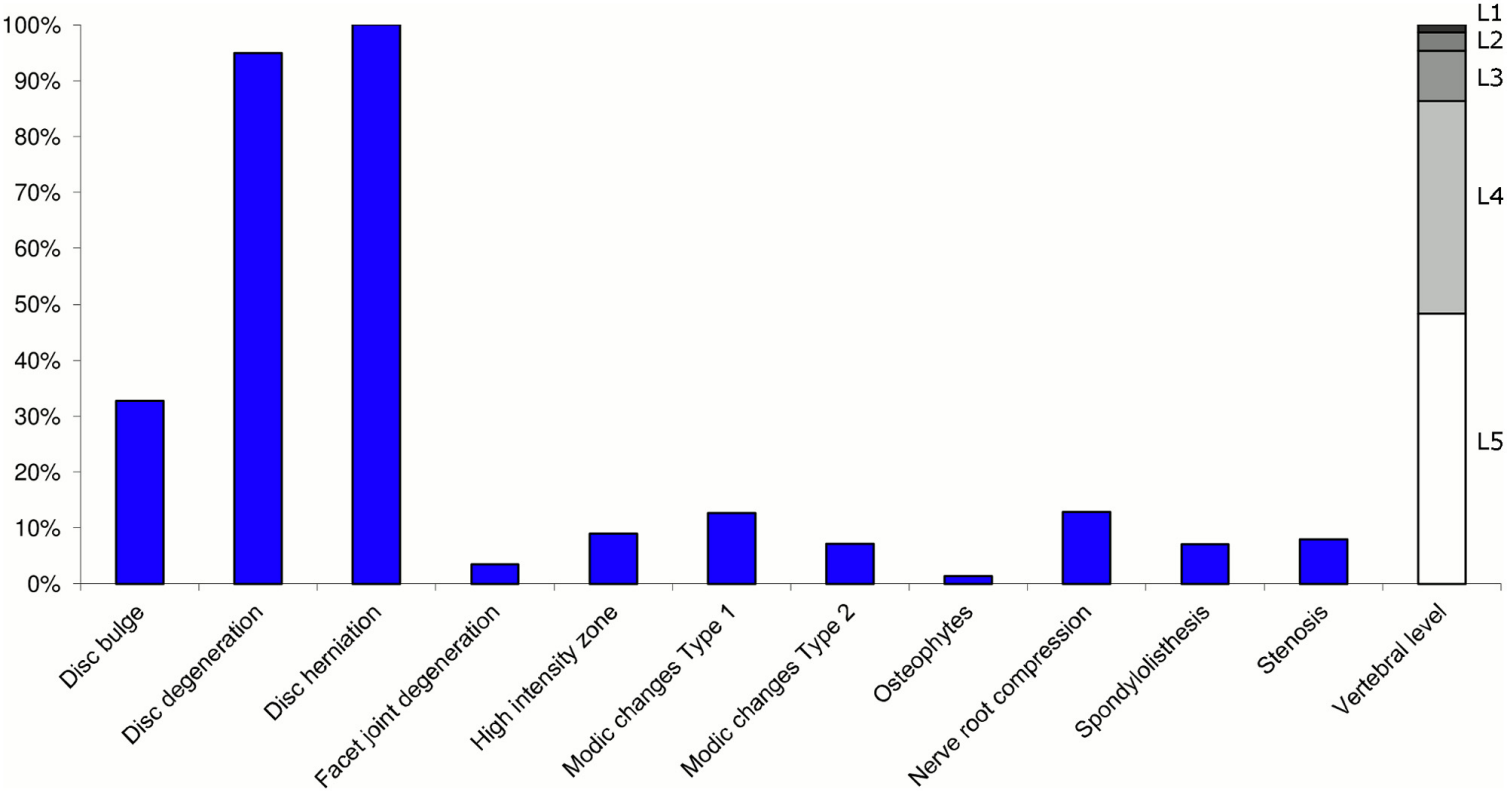
Prevalence of MRI findings within Cluster 1.3 in MRI_1. This cluster contained 10% of the 20,810 vertebral motion segments. (a) The vertical bars on the graph represent the proportion of vertebral motion segments for each of the MRI pathologies. (b) The mean age of the motion segments was 45 years (SD 13). (c) The vertebral level indicator shows the relative proportion of vertebral levels (L1 to L5) within the cluster.

**Fig 4:**
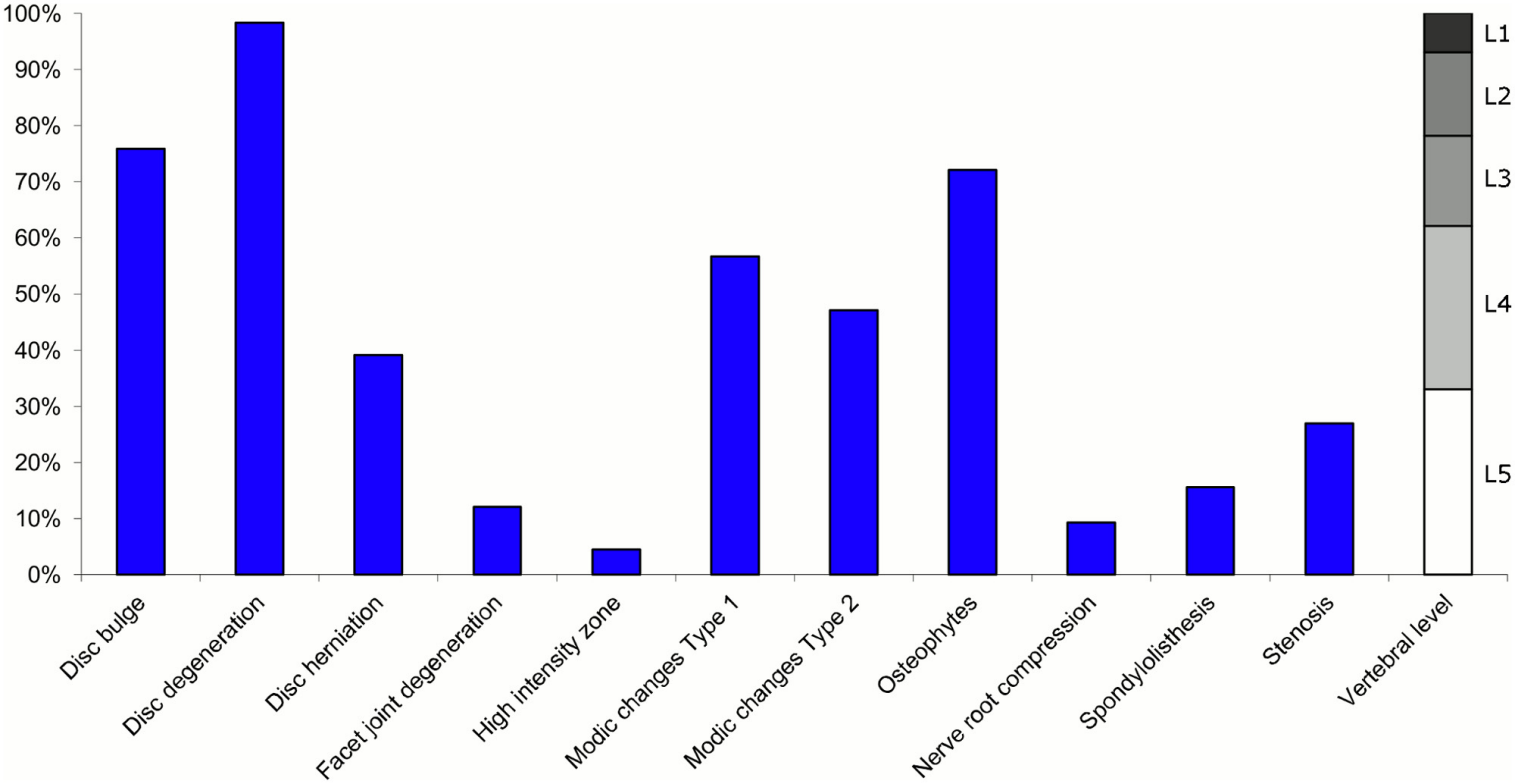
Prevalence of MRI findings within Cluster 1.4 in MRI_1. This cluster contained 4% of the 20,810 vertebral motion segments. (a) The vertical bars on the graph represent the proportion of vertebral motion segments for each of the MRI pathologies. (b) The mean age of the motion segments was 56 years (SD 12). (c) The vertebral level indicator shows the relative proportion of vertebral levels (L1 to L5) within the cluster.

**Fig 5:**
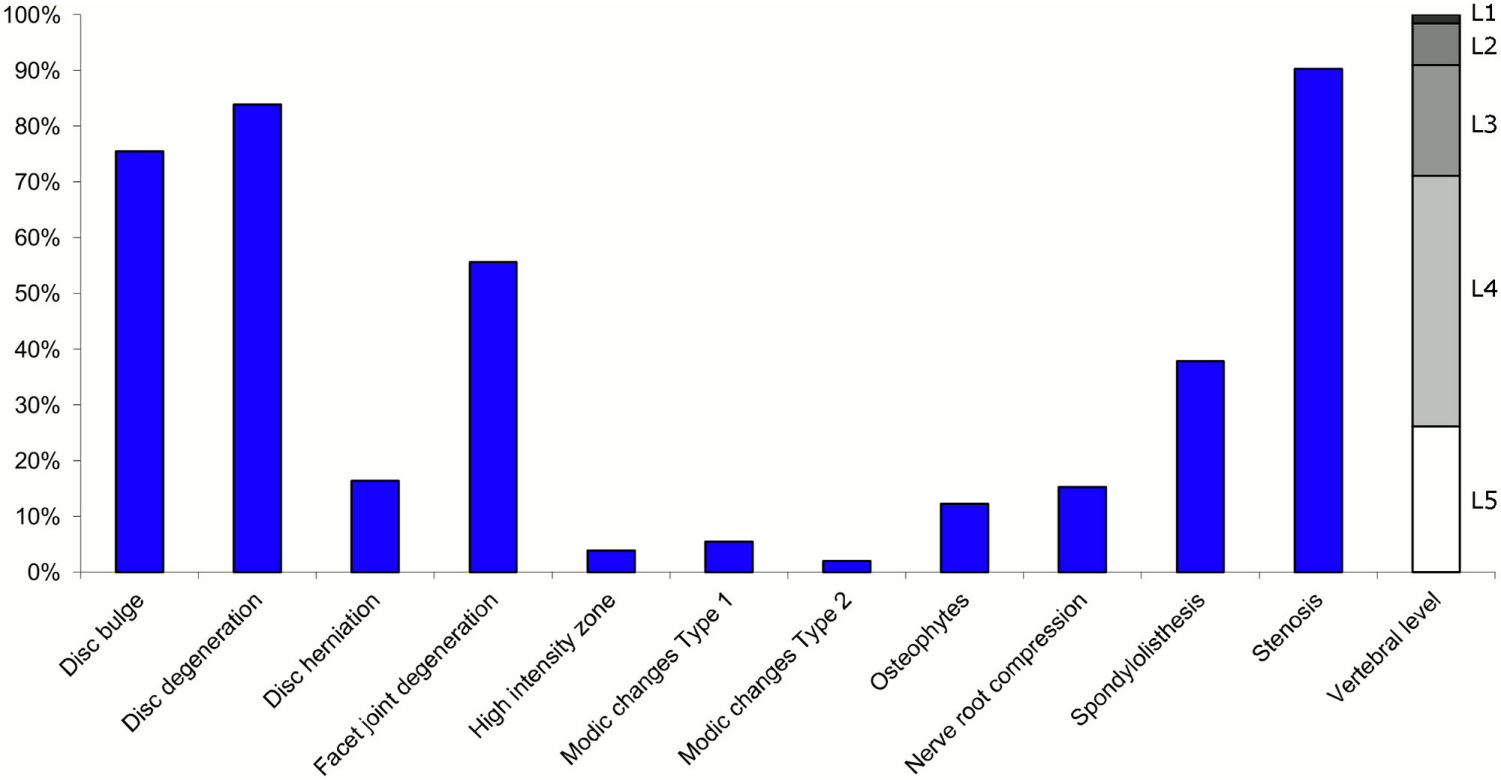
Prevalence of MRI findings within Cluster 1.5 in MRI_1. This cluster contained 3% of the 20,810 vertebral motion segments. (a) The vertical bars on the graph represent the proportion of vertebral motion segments for each of the MRI pathologies. (b) The mean age of the motion segments was 61 years (SD 14). (c) The vertebral level indicator shows the relative proportion of vertebral levels (L1 to L5) within the cluster.

**Fig 6:**
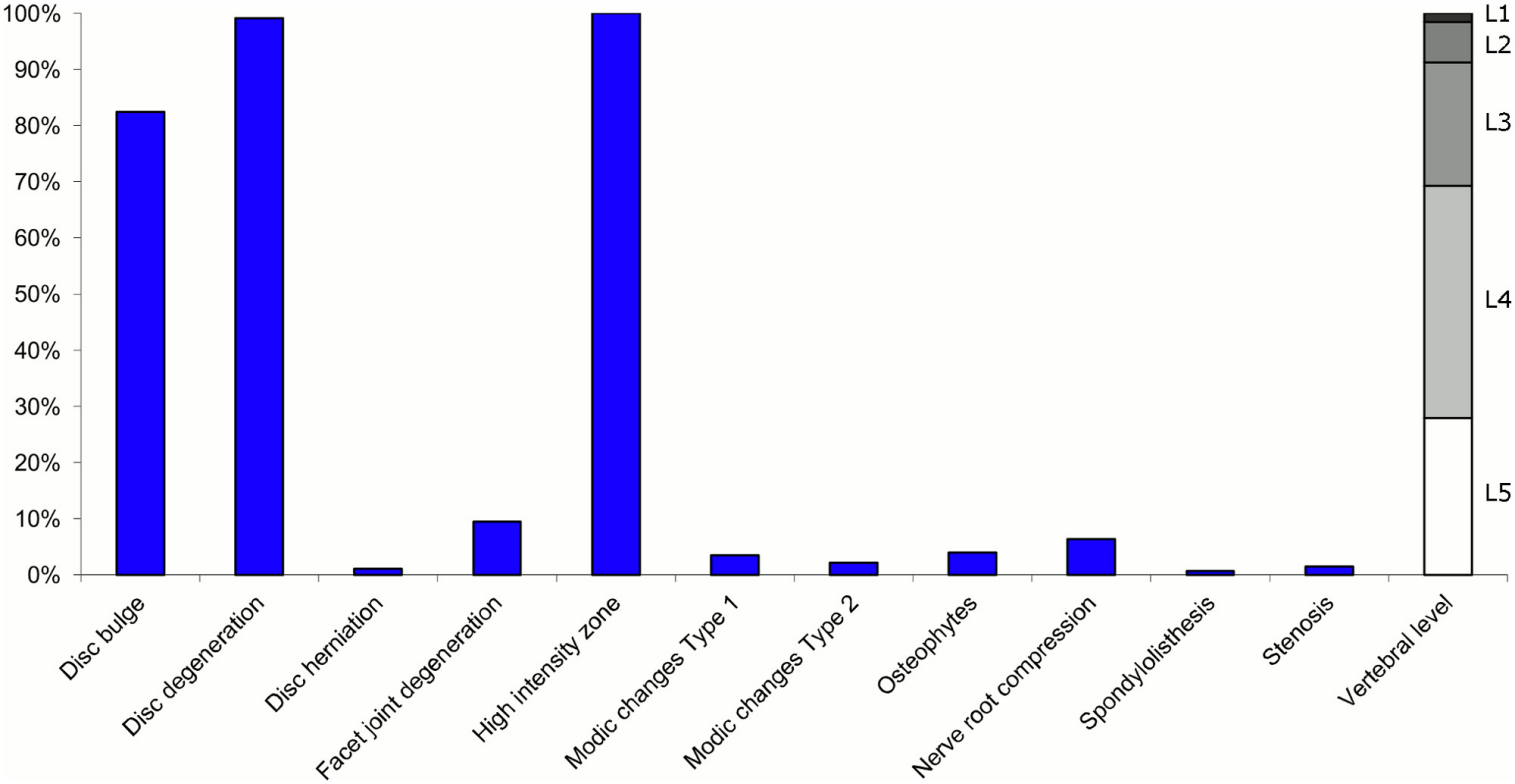
Prevalence of MRI findings within Cluster 1.6 in MRI_1. This cluster contained 2% of the 20,810 vertebral motion segments. (a) The vertical bars on the graph represent the proportion of vertebral motion segments for each of the MRI pathologies. (b) The mean age of the motion segments was 44 years (SD 10). (c) The vertebral level indicator shows the relative proportion of vertebral levels (L1 to L5) within the cluster.

**Fig 7:**
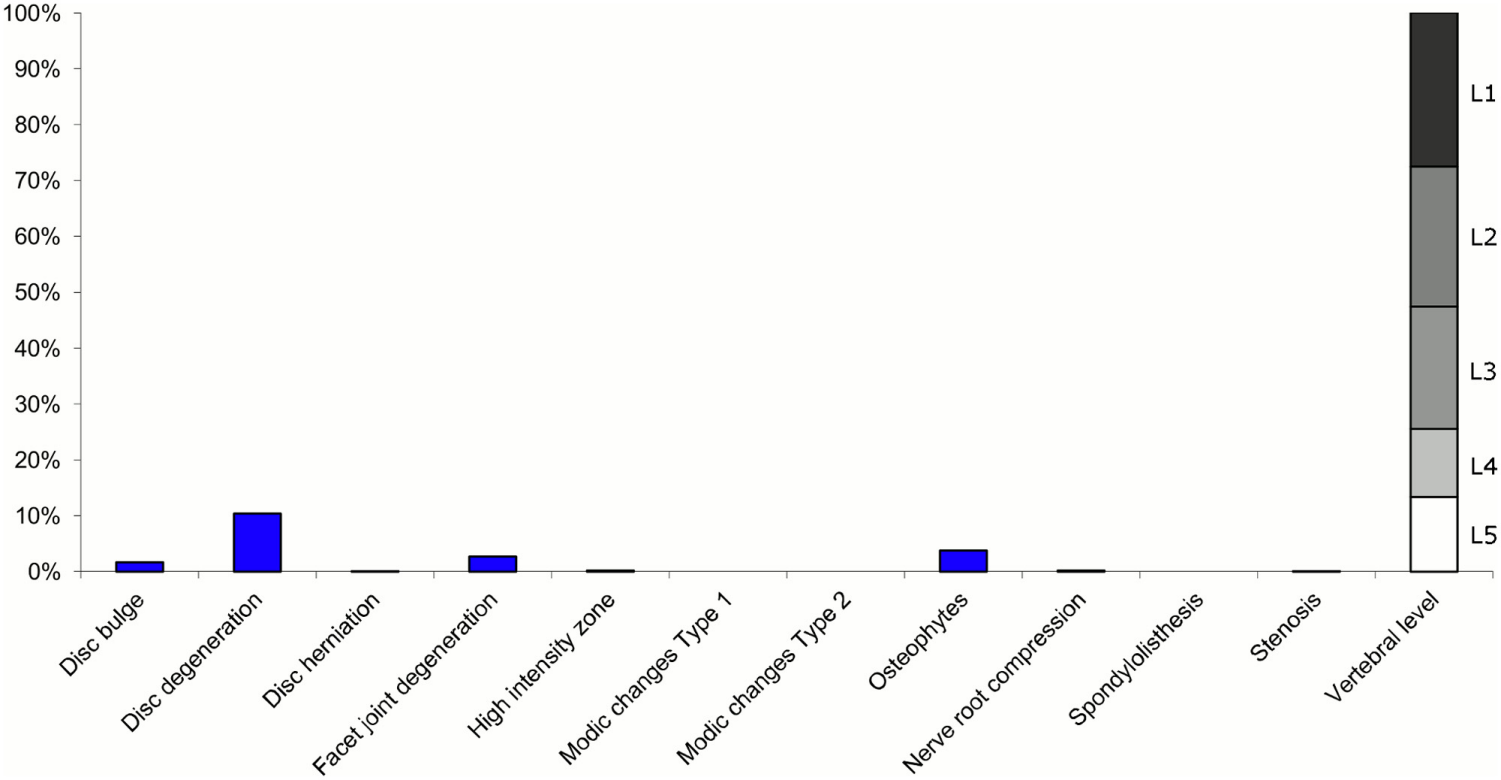
Prevalence of MRI findings within Cluster 2.1 in MRI_2. This cluster contained 57% of the 3,155 vertebral motion segments. (a) The vertical bars on the graph represent the proportion of vertebral motion segments for each of the MRI pathologies. (b) The mean age of the motion segments was 39 years (SD 10). (c) The vertebral level indicator shows the relative proportion of vertebral levels (L1 to L5) within the cluster.

**Fig 8:**
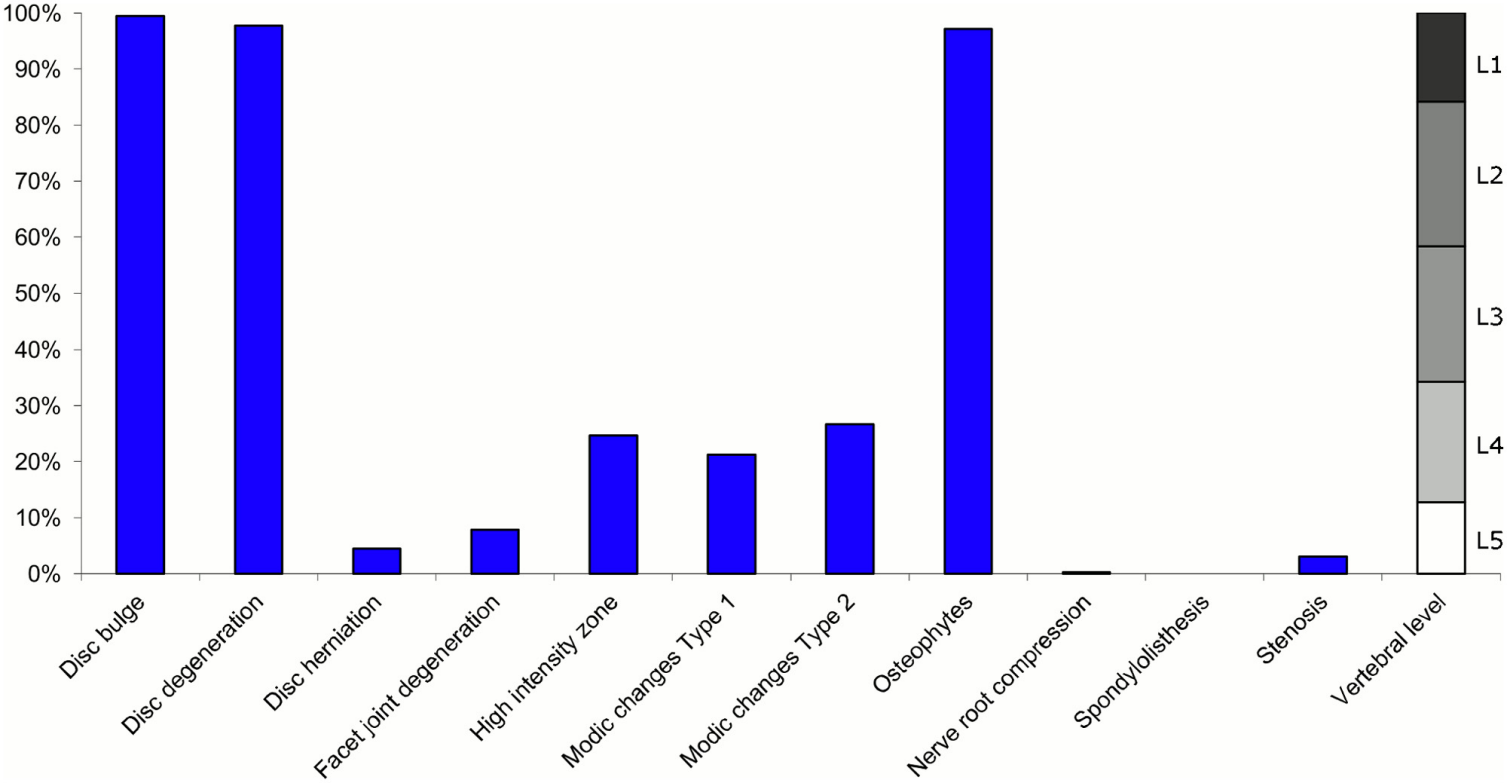
Prevalence of MRI findings within Cluster 2.2 in MRI_2. This cluster contained 11% of the 3,155 vertebral motion segments. (a) The vertical bars on the graph represent the proportion of vertebral motion segments for each of the MRI pathologies. (b) The mean age of the motion segments was 48 years (SD 9). (c) The vertebral level indicator shows the relative proportion of vertebral levels (L1 to L5) within the cluster.

**Fig 9:**
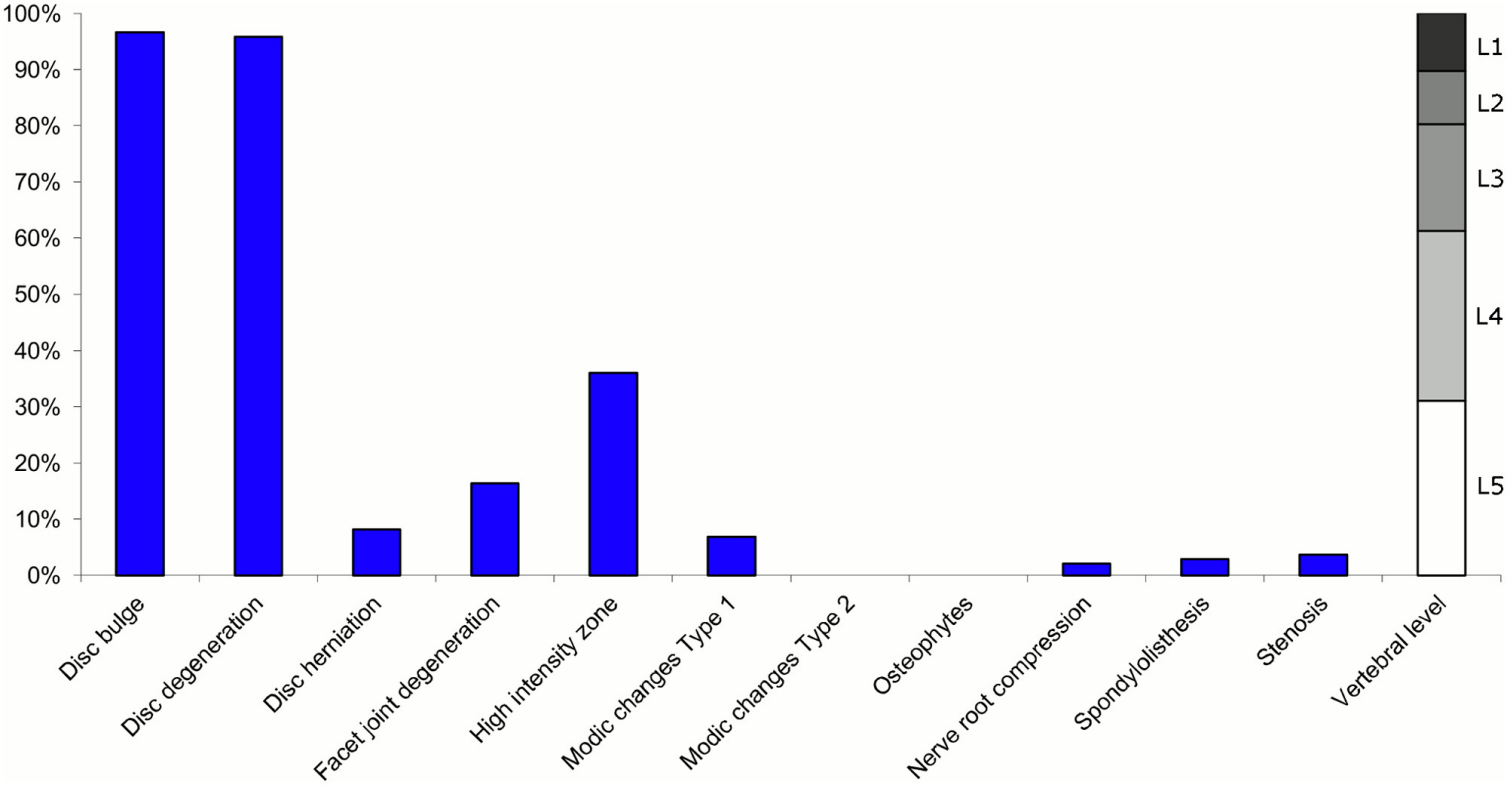
Prevalence of MRI findings within Cluster 2.3 in MRI_2. This cluster contained 12% of the 3,155 vertebral motion segments. (a) The vertical bars on the graph represent the proportion of vertebral motion segments for each of the MRI pathologies. (b) The mean age of the motion segments was 46 years (SD 11). (c) The vertebral level indicator shows the relative proportion of vertebral levels (L1 to L5) within the cluster.

**Fig 10:**
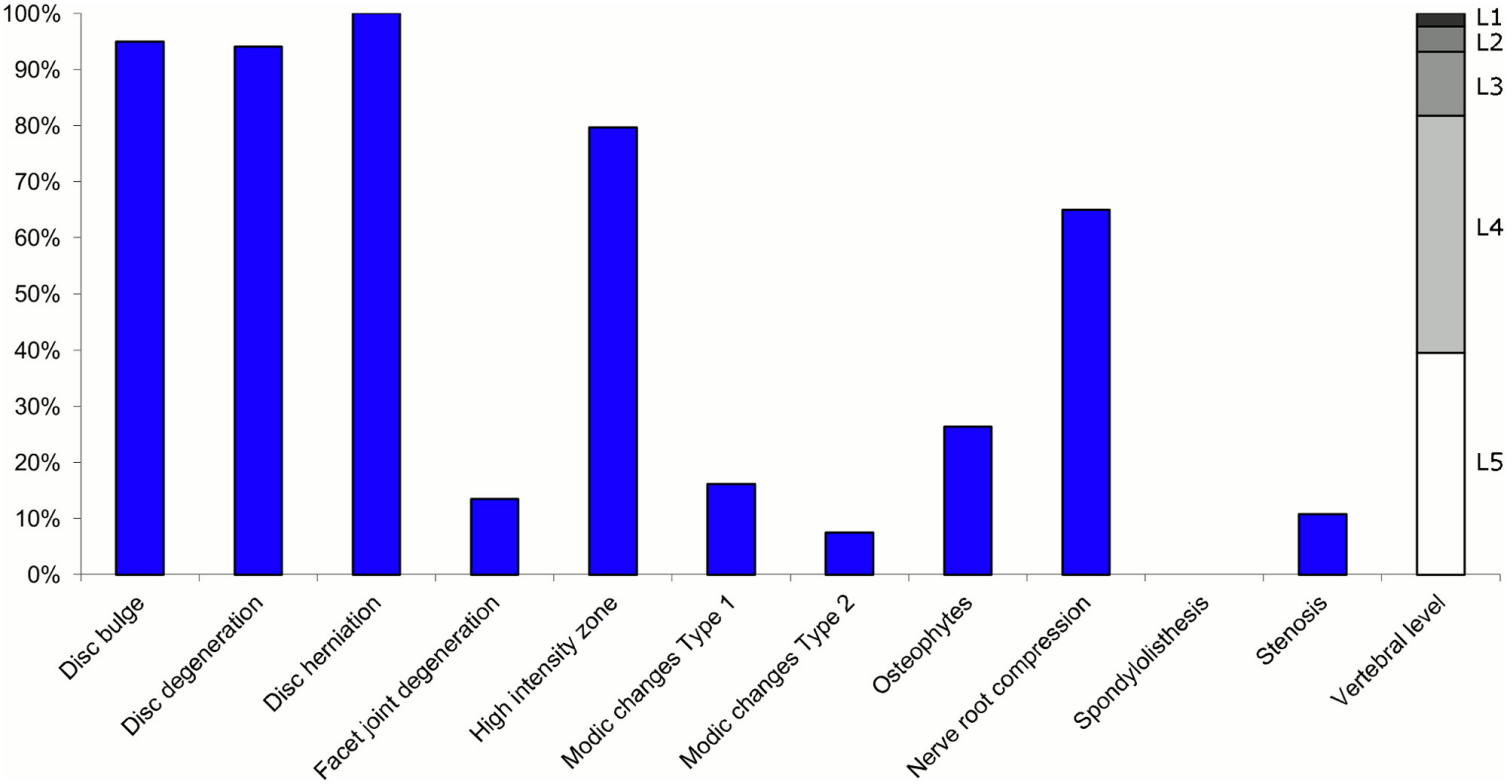
Prevalence of MRI findings within Cluster 2.4 in MRI_2. This cluster contained 11% of the 3,155 vertebral motion segments. (a) The vertical bars on the graph represent the proportion of vertebral motion segments for each of the MRI pathologies. (b) The mean age of the motion segments was 41 years (SD 10). (c) The vertebral level indicator shows the relative proportion of vertebral levels (L1 to L5) within the cluster.

**Fig 11:**
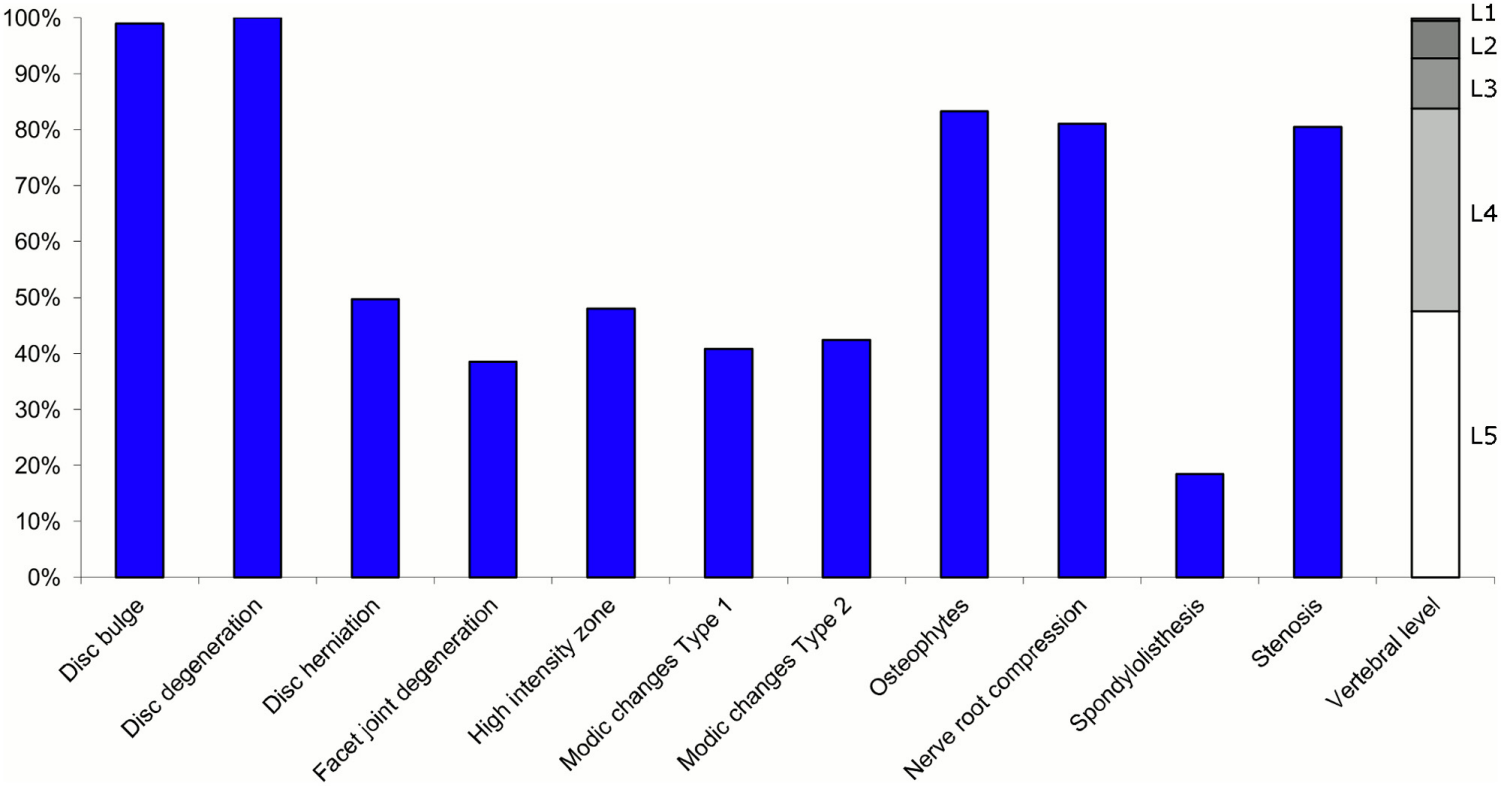
Prevalence of MRI findings within Cluster 2.5 in MRI_2. This cluster contained 6% of the 3,155 vertebral motion segments. (a) The vertical bars on the graph represent the proportion of vertebral motion segments for each of the MRI pathologies. (b) The mean age of the motion segments was 48 years (SD 11). (c) The vertebral level indicator shows the relative proportion of vertebral levels (L1 to L5) within the cluster.

**Fig 12:**
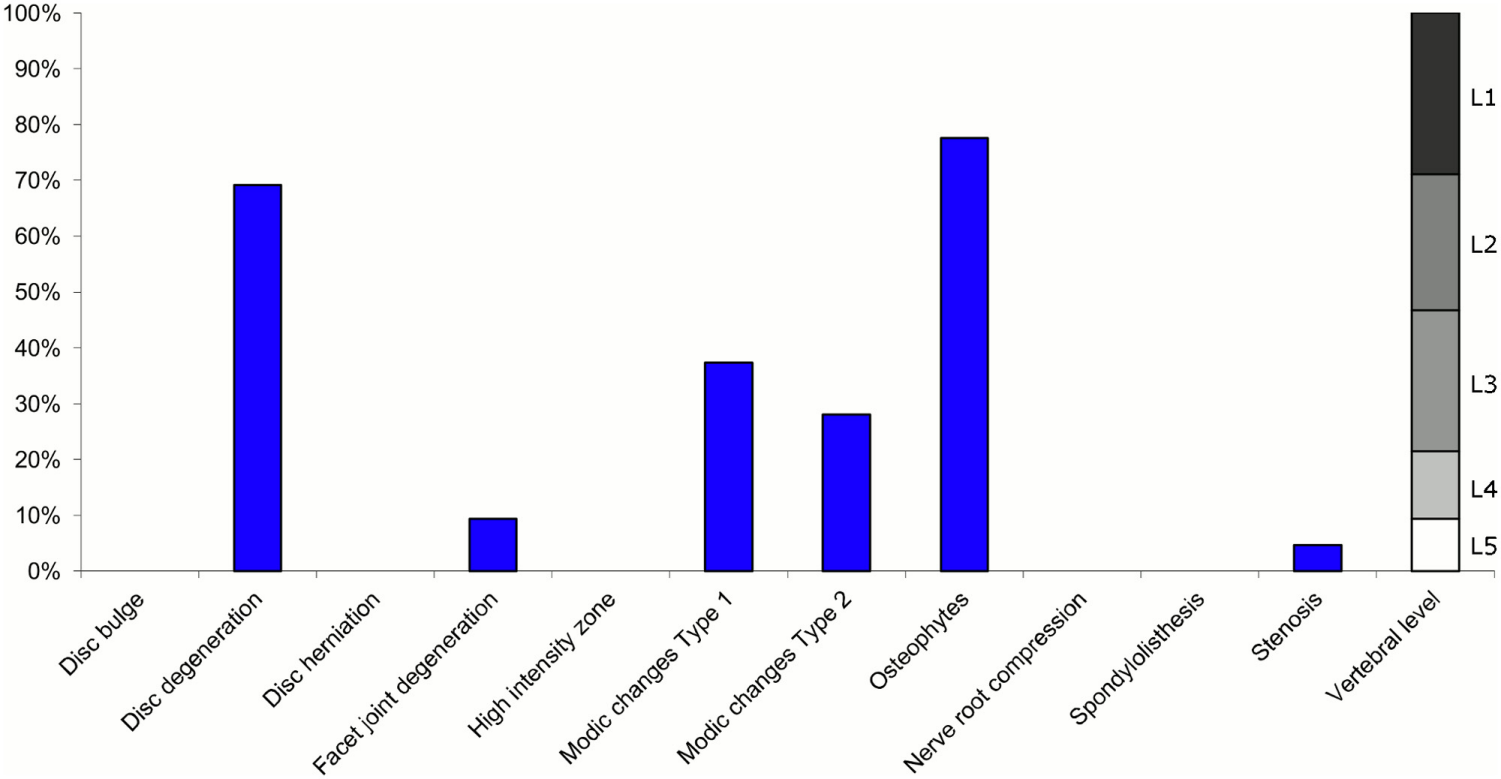
Prevalence of MRI findings within Cluster 2.6 in MRI_2. This cluster contained 3% of the 3,155 vertebral motion segments. (a) The vertical bars on the graph represent the proportion of vertebral motion segments for each of the MRI pathologies. (b) The mean age of the motion segments was 48 years (SD 10). (c) The vertebral level indicator shows the relative proportion of vertebral levels (L1 to L5) within the cluster.

Of the six clusters identified in MRI_1, the largest cluster was characterised by no or very few abnormal MRI findings and represented the normal, pre-degenerative state. This cluster (Cluster 1.1) contained 65% of the 20,810 motion segments and these were mainly located at the upper lumbar levels. The second largest (Cluster 1.2) contained almost 16% of the motion segments and was characterised by disc degeneration and bulges located at the three lower lumbar segments in three-quarters of the cases. The third largest cluster (Cluster 1.3) contained 10% of the motion segments and was characterised by disc degeneration and disc herniation located primarily at the two lowest lumbar levels. The three additional clusters (Cluster 1.4, 1.5 and 1.6), characterised by disc degeneration and additional findings such as Modic changes, facet joint degeneration, stenosis and HIZ, were infrequent, with each containing 4% or less of the motion segments.

Of the six clusters in MRI_2, the largest cluster (Cluster 2.1) was also characterised by no or very few abnormal MRI findings and contained 57% of the 3,155 motion segments. Three clusters each contained 11–12% of the motion segments. One of these clusters (Cluster 2.2) was characterised by disc degeneration, disc bulges, osteophytes, with approximately a quarter of the motion segments also having one or more of Modic changes Type 1, Modic changes Type 2 and HIZ. The MRI findings were almost equally distributed at all five lumbar levels. A second cluster (Cluster 2.3) contained motion segments with degeneration, bulges and some (one in three) HIZ. These MRI findings were at the three lowest lumbar levels in four out of five cases. A third cluster (Cluster 2.4) contained motion segments with disc bulges, disc degeneration, HIZ, disc herniations and root compression on the two lowest lumbar levels (four out of five cases). Of the remaining two clusters, one cluster (Cluster 2.5) contained 6% of the motion segments and displayed widespread degeneration at the lower vertebral levels, with all the MRI findings present. The last cluster (Cluster 2.6) contained 3% of the motion segments and displayed disc degeneration, osteophytes and Modic changes at the upper lumbar levels.

#### Hypothetical pathways of degeneration

**Fig 13:**
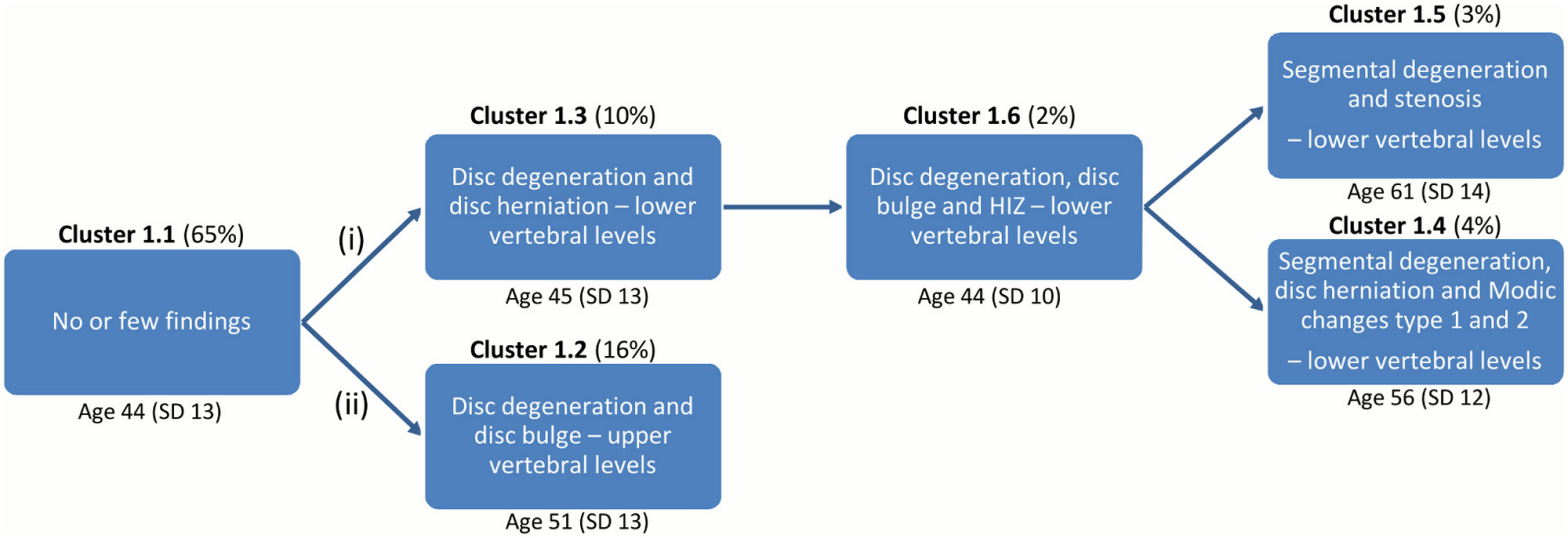
Hypothetical degenerative pathways of the vertebral motion segments in MRI_1. ‘Age’ is the average chronological age of the vertebral segments in each cluster and ‘SD’ is the standard deviation. The clusters were divided into the following two pathways: (i) Progressive stages of disc degeneration in the lower lumbar motion levels. (ii) Disc degeneration in the upper lumbar motion levels.

In MRI_1, two hypothetical degenerative pathways emerged from the content analysis of the clusters. The main pathway (Fig 13, pathway (i)) consisted of four clusters representing progressive stages of degenerative changes of the L4/5 and L5/S1 motion segments (62%-86% of the findings were present at those levels). This pathway had two different endpoints: one endpoint with Modic changes and the other endpoint with facet joint degeneration and spinal stenosis. The second pathway (Fig 13, pathway (ii)) had one cluster that was characterised by bulges and disc degeneration almost equally distributed across all five lumbar levels.

**Fig 14:**
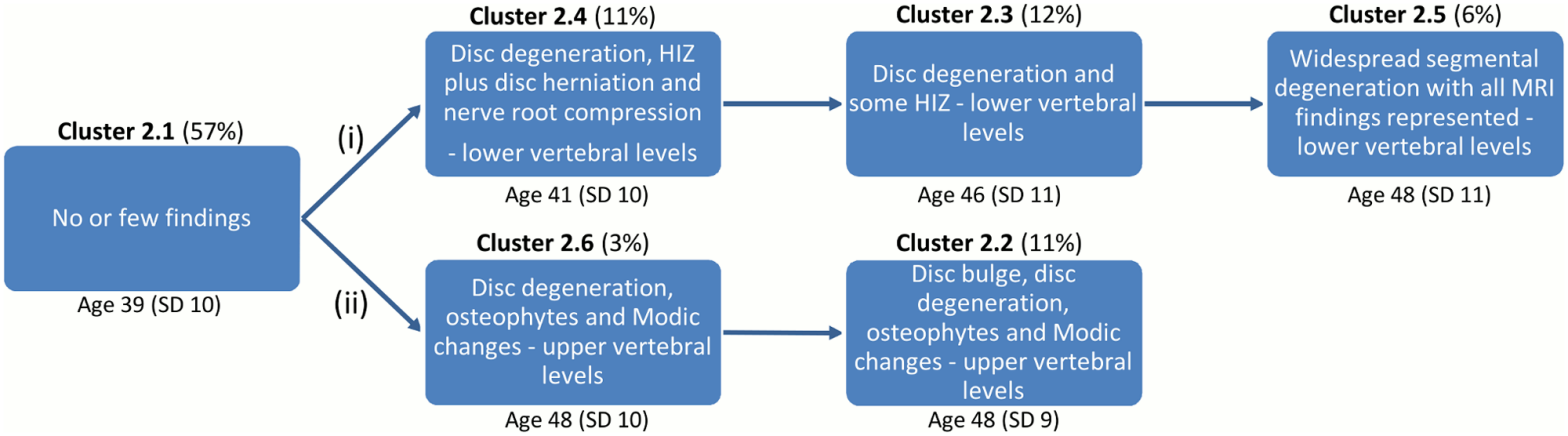
Hypothetical degenerative pathways of the vertebral motion segments in MRI_2. ‘Age’ is the average chronological age of the vertebral segments in each cluster and ‘SD’ is the standard deviation. The clusters were divided into the following two pathways: (i) Progressive stages of disc degeneration in the lower lumbar motion levels. (ii) Progressive stages of disc degeneration in the upper lumbar motion levels.

In MRI_2, two hypothetical degenerative pathways also emerged. One pathway (Fig 14, pathway (i)) contained three clusters that represented progressive stages of degenerative changes of the L4/5 and L5/S1 motion segments (80%-84% of the findings were present at those levels). The other pathway (Fig 14, pathway (ii)) had two clusters characterised by fewer MRI findings and primarily at the L1/2, L2/3 and L3/4 motion segments (66–79% of the findings were present at those levels).

Once the degenerative pathways had been categorised in a post-hoc analysis, the mean chronological age of the motion segments in each cluster was added to Figs 13 and 14. The chronological age within each pathway supported the notion of a developmental model, with the age broadly increasing along each pathway as the number of degenerative findings increased.

## Discussion

This study identified six clusters within each of the two samples (MRI_1 and MRI_2) and although the number of clusters found in the two samples was the same, the content of the clusters displayed some variability. This variability may be due to the different selection criteria, MRI descriptive method and prevalence of all MRI findings in the two samples. Clusters of MRI findings from lumbar spine motion segments were classified into biologically plausible hypothetical pathways of degeneration within each sample. A comparison of the degenerative pathways across the two samples of patients recruited from the same clinical setting showed an overall pattern characterised by an increasing prevalence of degenerative findings and a division between upper and lower lumbar levels.

In both samples, the largest cluster, containing more than half the motion segments, was comprised of segments that were almost completely without degenerative findings (normal motion segments). Also, in both samples, there was a trend towards the largest clusters of motion segments that did display degenerative findings being those with the fewest MRI findings (early degeneration), while the smaller clusters in each sample usually had more MRI findings (more advanced degeneration). In addition, the pathways with clusters containing a high prevalence of lower lumbar segments (L4/5, L5/S1) generally had more MRI findings than the pathways with clusters containing mostly upper lumbar levels (L1/2, L2/3 and L3/4). This differential distribution in the prevalence of clusters and the differential distribution of vertebral levels within each cluster are likely to be the result of an interaction between the biological age (degeneration) of the motion segments in each cluster and a tendency for degeneration to be accelerated in the lower lumbar spine due to biomechanical forces [7,12].

These biologically plausible hypothetical pathways of degeneration from both samples displayed an increasing number of ‘degenerative findings’ across clusters. This mapping of the ‘biological age’ of the motion segments was also broadly supported by the chronological age of the segments in each cluster, even though accelerated degeneration post-injury might have eroded the relationship between biological age and chronological age in some instances.

Although the content of the hypothetical pathways was not identical between the two samples, the overall pattern of degenerative pathways characterised by an increasing prevalence of degenerative findings was the same. In addition, in both samples, one pathway broadly described the degenerative findings located on the upper lumbar levels, while the other pathway described degenerative findings at the lower lumbar levels. Also, in the pathways that predominantly described the lower lumbar levels, the presence or absence of a lumbar disc herniation differentiated the clusters in the first part of the pathway in both samples, while spinal stenosis and/or Modic changes dominated the end stages in the pathways.

The patient characteristics between the cohorts differed, although both samples were recruited from the same setting. Patients in MRI_1 were a consecutive series referred for MRI based on the clinical criteria of individual clinicians, whereas MRI_2 were a consecutive series who met a study-specific set of inclusion criteria. This may account for the differences between the samples in the prevalence of individual MRI findings that are reported in our ‘Results’ section.

Differences between the samples in the MRI findings within clusters and the prevalence of the clusters may be due to a number of factors. In addition to these differences being likely to reflect contrasts in the clinical characteristics used to recruit people into the samples and divergences in the prevalence of individual MRI findings between the samples, they may also reflect variations in the radiological protocols used to report MRI findings. The MRI_1 sample was collected from narrative reports generated for clinical use only, where the cut-point for describing MRI findings was arbitrary, even though both radiologists aimed to perform descriptive radiology where they reported all abnormal findings regardless of their clinical significance. The MRI_2 sample was collected solely for research purposes and the images were evaluated according to a standardised and operationally defined MRI protocol.

Previous research supports a premise underlying our study, that is, that different clusters of MRI findings exist. In a recent study, Kanna et al.[14] analysed the patterns of lumbar disc degeneration in two groups of patients of the same age; (i) patients with persistent LBP and degenerative disc disease, and (ii) patients with acute disc prolapse (herniation). The authors concluded that these two groups were clinically and radiologically different with varying patterns, severity and extent of disc degeneration. In their sample, patients with acute disc herniation were less likely to have multilevel disc degeneration, including degeneration of all five lumbar disc levels, as compared with patients with persistent LBP. In both groups, degenerated discs were most often seen in the lower lumbar spinal levels, however, patients with LBP and degenerative disc disease also had a larger number of degenerative discs at the upper lumbar spinal levels compared with the patients with disc herniation. In other studies, Modic changes have previously been described as predominantly present at the lower lumbar levels and associated with the presence of other MRI findings such as herniation and disc degeneration[15,16] The prevalence of Modic changes seems to increase after the occurrence of disc herniation[17,18] implying some form of causal relationship.

This approach of using Latent Class Analysis with spinal MRI data is still novel, except for a ‘proof of concept’ study we performed using a more detailed sample and different Latent Class Analysis software[6]. We chose to use the Latent Gold software in the current study based on the results of a head-to-head comparison of three software packages (unpublished). However, others have used Latent Class Analysis to explore the relationship between MRI findings and clinical variables. Takatalo et al.[19,20] used this multivariable clustering approach to group multiple clinical variables from patients with LBP into five clusters which were then investigated for their association with individual MRI findings. Also, in a cross-sectional study of a birth cohort then aged 17 years, Beales et al.[21] used Latent Class Analysis to identify clusters of co-morbidities and health complaints associated with LBP. Four distinct co-morbidity clusters emerged and the relationships between co-morbidity cluster membership, health-related quality of life, and LBP were described. In contrast to our study where we clustered multiple MRI findings, the approach used in these other studies was to cluster clinical variables (LBP variables or co-morbidity) and relate these to single MRI findings or single clinical outcomes. Using data from people with LBP, others have used Latent Class Analysis to manage complex non-MRI data structures. In a method study, Kent and Kongsted found that Latent Class Analysis could identify clinical course patterns in patients with LBP using data collected by short text message (SMS messages)[22].

Our original ‘proof of concept’ study [6] showed that Latent Class Analysis could be used to identify clusters of MRI findings in a sample of people with LBP and those clusters could be grouped into biological pathways of degeneration with some face validity. The results of the current study confirm that the method can be applied to other samples with resultant degenerative pathways that also have some face validity. The results of the original study and the current study cannot be directly compared, as the original study used different MRI variables coded in different ways, and used different Latent Class Analysis software. However, the same overall pattern was observed of a large cluster containing ‘normal’ motion segments and divergent pathways containing clusters with increasingly prevalent degenerative findings. There is a need for further studies that extend this line of research before it will be possible to know the extent to which clusters and degenerative pathways are consistently reproduced.

The strength of this study is the relatively high quality of the samples. The MRI_2 was prospectively collected for research purposes only and had been rigorously described by an experienced research radiologist using a standardised data extraction protocol with high reproducibility. The MRI_1 was collected retrospectively and some variability of the description of the images would be expected, however, the inter-rater reliability of the quantification of the narrative reports was high[8]. Although not tested in this study, the radiologists who described the images have previously been tested in a research setting and been found to have good reproducibility of their MRI evaluations[11,23]. Also, the large size of the two samples used in this study enabled adequate power to identify clusters of MRI findings that have a low prevalence.

A weakness of this study is some remaining uncertainty about the reproducibility of the hypothesised biological pathways, as these two samples were not identical in clinical characteristics, prevalence of MRI findings, or MRI coding. One research design that could be used to investigate this would be to use split sampling of a large sample to examine reproducibility within a cohort, however while this would provide information of technical reproducibility, the results would not provide information about reproducibility across independent samples and settings. Knowledge about this broader reproducibility would require many samples from diverse settings. A further limitation is that research using different or additional MRI findings, such as the size and shape of the lumbar muscles and the degree of fat infiltration in the muscles may suggest different hypothetical degenerative pathways. Also, the clinical implications of clusters of MRI findings and degenerative pathways derived from them remains unknown and require further research.

## Conclusions

Six clusters with different content of MRI findings emerged in each sample and were organised into biologically plausible hypothetical pathways of degeneration with face validity. Although the content of the clusters displayed some variability across samples, the overall pattern was comparable, with a large cluster containing ‘normal’ motion segments and divergent pathways containing clusters with increasingly prevalent degenerative findings. If relatively stable clusters and pathways can be identified across multiple further samples, Latent Class Analysis could provide a new approach to investigating the relationship between degenerative pathways and clinically important characteristics, such as pain and activity limitation.
